# The effect of social connections on the discovery of multiple hidden food patches in a bird species

**DOI:** 10.1038/s41598-017-00929-8

**Published:** 2017-04-11

**Authors:** Zoltán Tóth, Beniamino Tuliozi, Davide Baldan, Herbert Hoi, Matteo Griggio

**Affiliations:** 1 Lendület Evolutionary Ecology Research Group, Plant Protection Institute, Centre for Agricultural Research, Hungarian Academy of Sciences, 1022 Budapest, Hungary; 2 Dipartimento di Biologia, Università di Padova, 35121 Padova, Italy; 3 Department of Animal Ecology, Netherlands Institute of Ecology (NIOO-KNAW), 6708 PB Wageningen, The Netherlands; 4 Konrad Lorenz Institute of Ethology, Department of Integrative Biology and Evolution, University of Veterinary Medicine of Vienna, 1160 Vienna, Austria

## Abstract

Social foraging is thought to provide the possibility of information transmission between individuals, but this advantage has been proved only in a handful of species and contexts. We investigated how social connections in captive flocks of house sparrows (*Passer domesticus*) affected the discovery of (i.e. feeding for the first time from) two hidden food patches in the presence of informed flock-mates. At the first-discovered and most-exploited food patch social connections between birds affected the order of discovery and presumably contributed to a greater exploitation of this patch. However, social connections did not affect discovery at the second food patch despite its close spatial proximity. Males discovered the food sources sooner than females, while feeding activity was negatively related to patch discovery. Age had no effect on the order of discovery. Birds that first discovered and fed at the food patches were characterized by higher level of social indifference, i.e. followed others less frequently than other birds in an independent context. Our findings provide experimental evidence for the importance of variable social connections during social foraging in house sparrow flocks, and suggest that social attraction can contribute differently to the exploitation of different patches when multiple food sources are present.

## Introduction

Animals often rely on social information when foraging: the presence of a conspecific individual at a food patch can transmit information about patch location, resource quality or accessibility^1–3^. The use of social information obtained from the observation of conspecifics’ behaviour^1, 4^ can lead to improved foraging opportunities and increased rate of food intake^5–8^, while also resulting in greater competition at the food source^9^. Models assuming the existence of social attraction among conspecifics however often do so without incorporating an underlying mechanism, simply expressing this effect as a function of the number of foragers present at the patch [e.g. refs 10–13, but see ref. 14]. While this can be true for individuals that are not socially associated with each other crowding at the same location (i.e. when animals aggregate at clumped and superabundant resources)^15^, in many species social groups are characterized by non-random associations between group-mates and in particular, members of the same social group may often follow each other or move together to a food source. In this case one individual exploiting a food patch can facilitate associated individuals to join that patch sooner than they or any non-associated group-mates ﻿otherwise﻿ would: resource discovery and exploitation are﻿ thus ﻿mediated by the presence of close social connections between certain group members^1, 16^.

The presence of variable social connections between group-mates can have far-reaching implications, because the social transmission of information, novel techniques or foraging skills is often not a function of the number of individuals present, but determined by complex interactions between individuals in the group^17–20^. Recent studies on such animal societies have provided evidence for the presence of cultural transmission of foraging innovations in mammals^19, 21, 22^, spread of experimentally induced foraging techniques and learning of foraging skills in birds^20, 23, 24^, discovery of prey patch locations and spread of foraging information in fish^25, 26^ and in insects^27^. Moreover, individual differences in the sensitivity to these social attraction effects or ‘social indifference’ [sensu 28] have also been proposed to play an inherent role in collective decision-making in animal aggregations [e.g. refs 2, 13, 29–31], similarly to the influential role of different “needs” due to individual differences in personal goals or motivation^28, 30^. However, the generality of these social effects, especially in less complex foraging scenarios but in the presence of multiple food sources, has been rarely addressed. We carried out our experiment on house sparrows, as it is an opportunistic and highly gregarious species^32^.

In this study we collected data on individual latencies to discover (i.e. first feed) from two hidden food patches in captive house sparrow flocks. In these flocks individuals did not have experience of these novel food sources, except for two informed flock-mates, which had been habituated to eat from similar hidden food patches, each marked with a colourful spot. These informed individuals were more knowledgeable about the location of the resource than other birds in the experimental flock, so their role was to speed up the discovery and exploitation of the hidden food patches during the trial. Using the first feeding events (i.e. patch discoveries), we investigated how the time of the discoveries predicted the extent of exploitation of the two food patches and how previously established social connections between flock-mates, measured in an independent context, and individual characteristics such as age, sex and feeding activity affected the order of discoveries among the naïve birds. Social connections were estimated from following networks constructed from the recorded following interactions between individuals and correspondence between patterns of association and food acquisition was tested using a modified version of network-based diffusion analysis (NBDA; [e.g. see in refs 1, 20, 24 and 33]). NBDA estimates the effect of social transmission on individuals’ acquisition of a trait or information based on the social connections previously measured in a social network, and quantifies how acquisition rate is accelerated when connected group-mates demonstrate the new trait. The applied variant of this analysis (OADA) has the advantage that it is insensitive to the shape of the baseline function (i.e. allows the baseline rate of acquisition to increase or decrease as the diffusion proceeds), and measures the relative rate at which individuals acquire the trait^34^. Aside from the following-based networks, we also generated homogeneous networks to model the situation when all individuals have equal opportunity to learn from each other [e.g. refs 24 and 26]. In this case, solely the increasing number of informed individuals may exert an acceleratory effect on the rate of acquisition at the food patches^34^. With this set-up, we aimed to explore the effect of social attraction on patch discovery in foraging house sparrows and test whether variable social connections influence individuals’ foraging decisions.

## Methods

### Study subjects

The experiment was carried out from October 2013 to April 2014 on a total of 108 house sparrows (54 males and 54 females) originating from a captive population at the Konrad Lorenz Institute of Ethology (University of Veterinary Medicine, Vienna, Austria)^35, 36^. Prior to the experiment, birds were kept in eight unisex outdoor aviaries (3.5 m × 3.5 m × 3 m; approx. 20 individuals per aviary; ‘initial flocks’ henceforward). Two weeks prior to the experiment, tutor flocks were formed from two groups of four individuals (2 males and 2 females randomly selected from the initial flocks) and were allocated into two outdoor aviaries (3.9 × 1.9 × 3 m; ‘tutor flocks’ henceforward). The experimental flocks, formed one at a time during the course of the study, consisted of ten adult individuals (5 males and 5 females) randomly chosen from the initial flocks (each individual was used only once) and were housed in an indoor aviary (2.8 × 2.7 × 2.1 m). In all flocks (initial, tutor and experimental), aviaries were equipped with a roosting tree, several perches and a water basin, and nestboxes for resting were also added to the initial and the tutor flocks. Commercial food for granivorous passerines was provided to all flocks according to the experimental design, which is described with further details in the Supplementary Information.

### Experimental procedure

#### Pre-training period

After the formation of the experimental flock, the pre-training period lasted for two days in which 250 g of food were provided every day on the central feeder at 8:00 am and removed at 18:00 pm. We recorded the foraging activity of the birds at the central feeder during the entire period when the food was present using the software iSpy 6 (video resolution 960 × 544 pixels, 7 frames per second). Individuals were unambiguously identified by the colour ring combination and the coloured marks on the head. We noted the time when birds arrived at the feeder and had access to food. Using these video recordings, two different foraging events were specified: following events (for a similar method see refs 37 and 38) and the total number of visits on the feeder. A following event occurred when an individual arrived at the feeding place and was followed by one or more group-mates within 5 seconds. The former individual was described as the ‘initiator’ and the latter(s) as the ‘follower(s)’. The total number of visits was defined as the sum of all foraging events by which an individual arrived at the central feeder (i.e. arrived alone, by following another group-mate or by arriving in a group without a specific initiator). This latter measure was used as a proxy for feeding activity during the analysis.

#### Training period

After the pre-training period, in the morning we randomly caught one male and one female from the experimental flock. These trainee individuals (the future informed birds in the hidden food patch trials) were inserted in the two tutor flocks by randomly assigning one sex to a specific tutor aviary (and as a consequence to a specific coloured marking). In the tutor aviaries trainee birds had access to food only under a marked box (out of two) on the ground, and had not previously encountered this novel food location. This habituation period lasted for several consecutive days (3.0 ± 0.47 day) during which the trainee birds visited the food source with the same frequency of the tutor birds (authors’ personal observations). This measure was used as an indication that the trainee birds foraged at the box with food similarly to tutor birds. We also waited until we observed the trainee birds visiting the marked box on their own initiative, not only by following the tutors. For the rest of the birds in the experimental flock, food was always provided *ad libitum* on the central feeder throughout this period, thus only the trainee birds had experience in feeding from a food patch under the box.

#### Trials

Once both trainee individuals foraged at the food source hidden under the marked boxes at the same frequency as the tutor birds, the trial started on the following day. In the morning the central feeder from the experimental aviary was removed and the two trained birds were re-introduced into the experimental flock. The four cardboard boxes were removed and under two of them 52.14 ± 3.35 g of millet spray were anchored to one of the inner sides. The boxes were then placed inside the aviary; as the positions of the food-hiding boxes were *a priori* assigned, the two webcams were already positioned in front of them. The coloured markings were added and randomly associated to a box with food. All birds were familiar with the boxes as these were added to the aviary of experimental flocks from the beginning of the pre-training period, but food was placed under two of such boxes only when the trial started, with a light blue or magenta marking placed on the top of each box serving as a visual cue for the informed birds. Informed birds and the rest of the experimental flock were food deprived for the same time period (from 8:00 until the video recordings in the experimental aviary started). Hidden food contained inside the two boxes was the only food source available during the trial. Once the food was placed inside the aviary, the trial and the video recording lasted until 18:00. As the video recordings did not start exactly at the same time in all flocks, the maximum duration of the trial was set to 30420 seconds (shortest duration among the flocks) to standardize the time frame for patch discovery.

Video recordings at the hidden food sources were collected during the trial when food was present under the two boxes in the experimental flock. The two cameras were time-synchronized and set to record when movement in front of the cameras was detected by using the software iSpy 6 (with the same settings as above). Using the colour ring combination and the coloured marks on the head, we identified each individual on the video recordings that ate directly from the food source and measured its latency to feed for the first time at each food patch (‘latency to feed’). For each food patch, we also recorded the first time when an individual approached it by hopping toward the millet spray and visually inspecting it (‘first approach’), and the number of aggressive interactions between birds during the trial. At the end of the day the remaining amount of food at both patches was measured as the difference in weight of the millet sprays before and after the trial at each patch.

### Constructing social networks

We used following events collected in the pre-training period as direct interactions between individuals to characterize social connections and construct directed weighted social networks. Nodes in these following networks represented individuals in the flock, and edges represented following rates, i.e. the number of followings per hour, which were calculated as the total number of occasions when one individual followed another divided by the duration of the trial in hours (which is not necessarily the same as the number of followings per hour by which the other bird followed the first one). We used these following networks test in the NBDA to test whether social transmission of information about the hidden food patches follows the pattern of associations in the house sparrow flocks. We also calculated in- and out-strength network metrics for each individual from these following networks, which denote the frequency of being followed by other flock-mates (per hour) and the frequency of following others in the flock ﻿(per hour), respectively^38, 39^.

### Statistical analysis

We used R 3.3.2 for all statistical calculations^40^. We applied Approximative Wilcoxon-Pratt Signed-Rank Tests, Approximative Wilcoxon-Mann-Whitney Tests and Approximative Spearman Correlation Tests with 19999 iterations from the ‘coin’ R package^41^ to estimate between- and within-flock differences and correlations; ‘flock’ was used for stratification in all two-sample comparisons and correlation tests. Directed weighted following networks were constructed and individuals’ in- and out-strength were calculated using the ‘tnet’ R package^42^. We applied a modified version of the order of acquisition diffusion analysis (OADA) variant of NBDA^34, 43^, which is fitted to the collected data on the order in which individuals acquire a behavioural trait relative to other naïve individuals. In the modified OADA, the computation of the social transmission parameter did not differ from that of the original OADA^34^, but instead of using standard Cox proportional hazard models we applied Cox mixed-effect proportional hazard models during optimization and model fitting. This subtle change in the calculation routine allowed us to include the necessary random term, i.e. ‘individual identity’ nested into ‘flock’, into the models for the investigation of trait acquisition at the two patches within each flock. Also, we combined the acquisition diffusions into a single dataset, which allowed us to take different baseline rates at the two patches into account through stratification of the data (for more details, see Supplementary Information). Since this method preserves information about which individual comes from which diffusion, we could test whether or not social transmission rates differed at the two patches. To fit separate parameters for social transmission for different tasks, we used a specific argument (sParam in the NBDA context) and fitted models in the following scenarios: no social transmission at either patch (i.e. asocial models), same rate of social transmission at both patches, different rates of social transmission at the two patches, social transmission only at the first-discovered patch, social transmission only at the second-discovered patch (‘model categories’). Into the models we incorporated the presence of informed birds, which means that social connections of informed individuals to naïve flock-mates created opportunity for social transmission to operate already at the very first discovery event in a flock. We also added individual transmission weights to each bird to control for the possibility that individuals may perform the acquired trait (i.e. feeding from a hidden food patch) at a different rate in their flocks due to their differences in foraging activity; weights were calculated as the total number of visits to the central feeder in the pre-training period scaled to the maximum value in the given flock. We tested the effects of individual characteristics such as feeding activity, sex and age; the latter two were previously found to affect individuals’ position in following networks in house sparrows^38^. Social connections were based on either the constructed following-based or homogeneous networks (i.e. all connections set to 1). We tested all potential combinations of the above scenarios, explanatory variables and type of social connections, and then ranked the models according to their predictive power using Akaike Information Criteria corrected for small sample sizes (AICc^44^) and corresponding Akaike weights^44, 45^ (Table S2). Preliminary analysis of OADA models fitted with following-based networks separately at the two patches indicated a stronger support for the multiplicative models (ref. 34, Table S3), thus in our final analysis social and asocial acquisition processes could interact multiplicatively (i.e. only multiplicative models were fitted). Conditional 95% confidence intervals for the social transmission rate and the explanatory variables were calculated using profile likelihood technique^46, 47^. We based our inference on these estimations in the ‘best models’ set (within 4 ΔAICc with the best-fitting model), as we could not calculate conditional standard errors for model-averaging from the numerical estimate of the Hessian matrix in those models which contained different transmission parameters for the two food patches. More details about the applied analysis, together with the constructed R script, are provided in the Supplemental Information. NBDA was performed using the code provided on The Laland Lab’s website (NBDA code v1.2.13; http://lalandlab.st-andrews.ac.uk/freeware.html) with the modifications detailed above.

### Ethics Statement

Capture, housing and handling of birds were in accordance with the relevant Austrian laws and were licensed by the government of Vienna (MA 22) license number 424/2011. The experiment reported in this study complies with current laws on animal experimentation in Austria and the European Union. This study was approved by the institutional ethics committee (University of Veterinary Medicine, Vienna) and the national authority according to 8ff of Law for Animal Experiments Tierversuchsgesetz - TVG, licence number GZ 68.205/0220-II/3b/2012.

## Results

**Figure 1:**
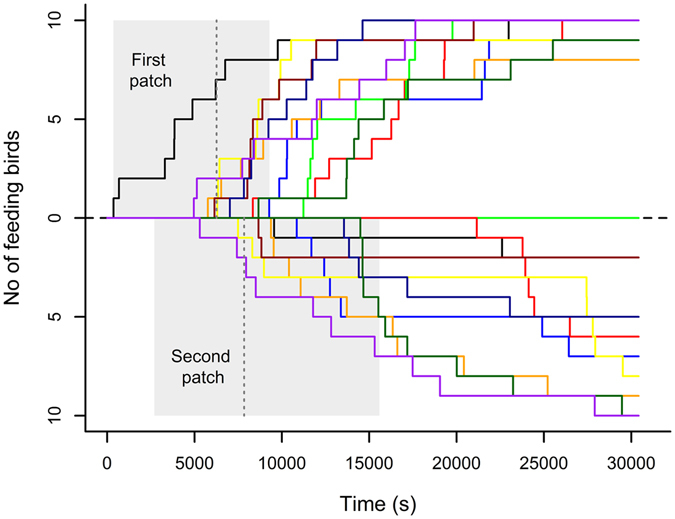
Diffusion curves showing the latency of individuals to feed from the two hidden food patches in the flocks. Each coloured line represents one flock, and the same colour denotes the same flock at the first-exploited patch (upper half of the panel) and the second-exploited patch (lower half of the panel). Time to first approach is indicated by a grey bar at each patch, with the dashed lines showing the mean values.

In 9 out of 10 flocks birds fed from both hidden food patches during the trials, but the two patches were exploited differently (Table 1): from the first-approached patch (Approximative Wilcoxon-Pratt Signed-Rank Test: *Z* = −2.09, *P* = 0.038) individuals obtained food earlier than from the other patch; this was true for those birds that fed first from this patch (i.e. first-feeders; *Z* = −2.80, *P* = 0.002) and also in the case of an average bird (*Z* = −2.55, *P* = 0.009). By the end of the trial more birds fed from the first-discovered patch (*Z* = 2.15, *P* = 0.036; Fig. 1) and more seeds were taken by the end of the trial from this patch than from the second-discovered patch (*Z* = 2.40, *P* = 0.014). During feeding, the frequency of aggressive interactions was also higher at the first than at the second patch (*Z* = 2.14, *P* = 0.028). There was a strong and significant negative correlation between the average time of first feeding (with birds that did not eat from one of the two patches being excluded) and the amount of food loss at the first patch (*r*_S_ = −0.71, *N* = 10, Approximative Spearman Correlation Test with ‘flock’ used for stratification: *Z* = −2.13, *P* = 0.026), but this relationship was weak and non-significant at the second patch (*r*_S_ = −0.23, *N = 9*, *Z* = −0.66, *P* = 0.555). These findings indicate that although uncertainty regarding patch location was likely to decrease over time as both patches became utilized by more and more individuals, first-feeding latencies predicted the extent of exploitation at the first, but not at the second patch. Also, the first-approached food patch was exploited to a higher extent by more individuals under a higher competition regime by the end of the trial compared to the second-approached patch despite their close spatial proximity (~0.5–1.5 m).

**Table 1:** Mean ± SD of the investigated parameters at the two food patches in the house sparrow flocks.

	First-discovered food patch	Second-discovered food patch
Time of first approach (s)	6263.9 ± 2807.23	7838.9 ± 4039.47
Time of discovery by the first-feeder birds (s)	6807.1 ± 2948.77	13490 ± 8591.43
Time of discovery by an average bird (s)	11554.53 ± 3424.98	16544.33 ± 4517.11
Number of birds discovered the patch	9.5 ± 0.71	5.9 ± 3.57
Amount of seed taken (g)	36.5 ± 10.44	14.66 ± 10.95
Frequency of aggressive interactions	33.7 ± 39.86	9.8 ± 13.60

In the OADA we found that the order of discovery was affected by the presence of variable social connections: models fitted with following-based networks had a 2.67 × higher overall support than those fitted with homogeneous networks (72.78% vs. 27.22%; Table S3). Asocial models had very low relative support in general (<0.01%; Table S3). In the ‘best models’ set (i.e. those within 4 ΔAICc with the best-fitting model; 69.11% overall support), the social transmission parameter at the first patch was estimated to be higher than zero in all models, while at the second patch it was either constrained to or not different from zero (Table 2). This result indicates that social transmission of information between naïve birds and any of those individuals that already discovered the patch could occur at the first-discovered food patch if these individuals were connected even only by a few followings within an hour (or by a single following per hour if the bird that already discovered the patch had the highest transmission weight in the flock). A likelihood ratio test between the best-fitting model and its corresponding asocial model also indicated a significant effect of social transmission at this patch (χ^2^_1_ = 29.71, *P* < 0.001). Both sex and feeding activity had a significant effect on the order of patch discovery in all models in the ‘best models’ set (Table 2), implying that males discovered the food patches sooner than females and more active birds discovered the food patch later. This latter finding may indicate that the measured feeding activity was rather related to the number of ‘feeding alone’ events in birds than to their tendency to forage in groups. Age was found to have a negligible effect on patch discovery in these models (Table 2).

**Table 2:** Model parameter estimates and their conditional profile likelihood 95% confidence intervals in the two best supported categories of models fitted with following-based networks.

Model category	AICc	ΔAICc	*w*_Akaike_	Parameter estimates [95% CI]	*s*_2_	Age	Sex	Feeding activity*
				*s*_1_				
Social transmission only at patch 1 (support: 45.91%)	**1037.81**	**0.70**	**0.16**	**1.633 [0.550–5.161]**	**constrained to 0**	**0.182 [−0.112–0.471]**	**0.415 [0.078–0.753]**	**−0.004 [−0.005–0.002]**
	1050.37	13.26	0.00	0.572 [0.185**–**1.811]	constrained to 0	0.284 [**−**0.083**–**0.586]	0.366 [0.032**–**0.707]	**—**
	***1037.11***	***0.00***	***0.23***	***1.571 [0.527*** **–** ***4.986]***	***constrained to 0***	***—***	***0.431 [0.095*** **–** ***0.768]***	***−0.004 [−0.005*** **–** ***0.002]***
	**1041.08**	**3.97**	**0.03**	**1.191 [0.402–3.703]**	**constrained to 0**	**0.204 [−0.089–0.492]**	**—**	**−0.003 [−0.005–0.002]**
	1052.56	15.44	0.00	0.442 [0.136**–**1.438]	constrained to 0	0.286 [**−**0.039**–**0.585]	—	—
	1050.81	13.69	0.00	0.564 [0.171**–**1.890]	constrained to 0	—	0.394 [0.057**–**0.739]	
	**1040.80**	**3.69**	**0.04**	**1.090 [0.367–3.540]**	**constrained to 0**	**—**	**—**	**−0.004 [−0.005–0.002]**
	1053.57	16.46	0.00	0.411 [0.112**–**1.452]	constrained to 0	—	—	—
Different social transmission rates at the two patches (support: 26.17%)	**1039.00**	**1.89**	**0.09**	**1.835 [0.626–5.852]**	**0.120 [0–0.601]**	**0.169 [−0.125–0.459]**	**0.446 [0.109–0.784]**	**−0.004 [−0.006–0.002]**
	1052.56	15.44	0.00	0.572 [0.185**–**1.811]	0 [0**–**0.188]	0.284 [**−**0.083**–**0.586]	0.366 [0.032–0.707]	—
	**1038.05**	**0.94**	**0.14**	**1.803 [0.613-5.760]**	**0.137 [0–0.644]**	**—**	**0.464 [0.129–0.800]**	**−0.004 [−0.006–0.003]**
	1042.89	5.78	0.01	1.251 [0.425**–**3.908]	0.061 [0**–**0.424]	0.197 [**−**0.096**–**0.485]	—	**−**0.004 [**−**0.005**–**0.002]
	1054.71	17.59	0.00	0.442 [0.136**–**1.438]	0 [0**–**0.161]	0.286 [**−**0.039**–**0.585]	—	—
	1052.96	15.84	0.00	0.564 [0.171**–**1.890]	0 [0**–**0.196]	—	0.394 [0.057**–**0.739]	—
	1042.43	5.32	0.02	1.163 [0.395**–**3.798]	0.075 [0**–**0.457]	—	—	**−**0.004 [**−**0.006**–**0.002]
	1055.69	18.58	0.00	0.403 [0.112**–**1.453]	0 [0**–**0.168]	—	—	—

*As feeding activity was measured as the total number of visits at the central feeder, the estimated decrease in log odds of discovery corresponds to one unit increase in feeding activity.

The model with the lowest AICc is written in italics, while the ‘best models’ set (within 4 ΔAICc) on which we based our inference is written in bold. Other model categories (same social transmission rate at both patches, social transmission only at the second patch, no social transmission at either patches) had very low overall support (≤0.70%). AICc values and corresponding Akaike weights of all models (both fitted with following-based and homogeneous networks) are shown in Table S3.

**Figure 2:**
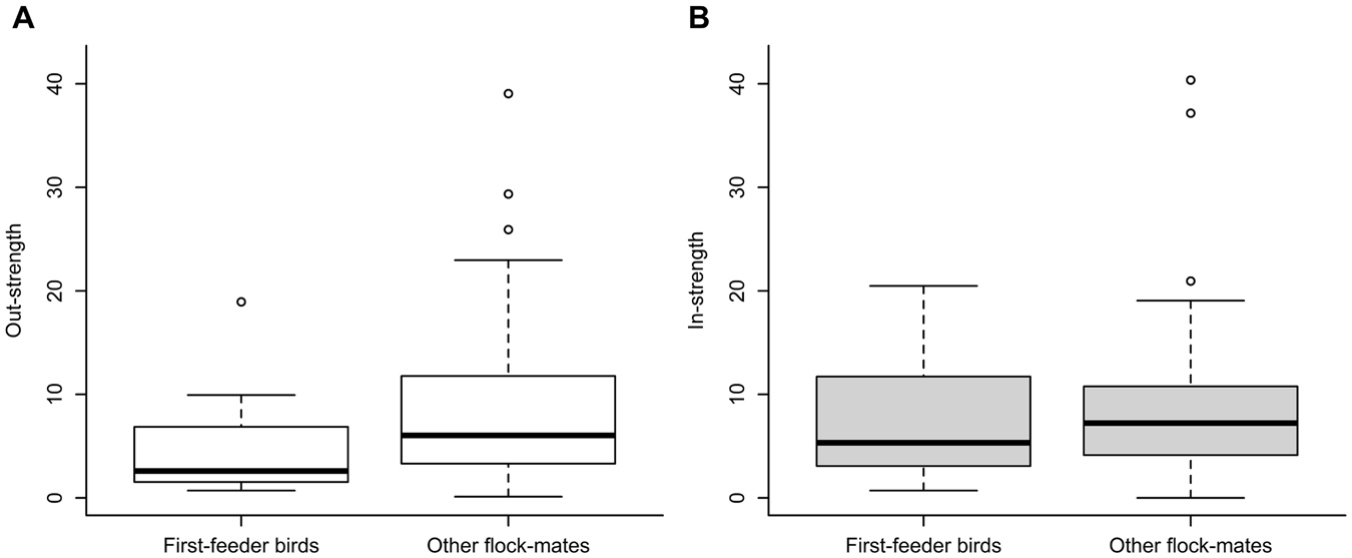
Differences in out-strength and in-strength between the first-feeder birds and other birds in the flocks. Out-strength (**A**) was calculated from the number of followings per hour when the focal bird followed a flock-mate to the feeder during the pre-training period, whereas in-strength (**B**) was derived from the number of followings per hour during which the focal bird was the initiator individual. These metrics were derived from the constructed following networks and reflect individuals’ social position (in terms of their tendency to follow others and elicit followings from others, respectively) in their flock. Horizontal lines are medians, the boxes and the whiskers show the interquartile ranges and the data ranges, respectively.

Being informed did not predict to be a first-feeder more frequently than expected by chance (6 first-feeder informed birds out of 19 events [the second patch remained unexploited in one flock]; Binomial test: *P* = 0.246), although informed individuals gained access sooner to the first (informed birds: 9765.5 ± 3193.91 s, other flock-mates: 13760.66 ± 7730.91 s, Approximative Wilcoxon-Mann-Whitney Test with ‘flock’ used for stratification: *Z* = 2.67, *P* = 0.008), but not to the second patch (informed birds: 24796.75 ± 9992.03 s, other flock-mates: 23467.56 ± 9843.88 s, *Z* = −0.67, *P* = 0.503), compared to other birds. The coloured marks did not have any effects whatsoever on the time of patch discovery during the trial (data not shown). Sex of the informed birds did not affect the average latency of flock-mates to feed from a patch (male informed birds: 15895.87 ± 5635.65 s, female informed birds: 19325.18 ± 7493.21 s, Approximative Wilcoxon-Pratt Signed-Rank Test: *Z* = −0.87, *P* = 0.433). However, first-feeder birds, i.e. individuals that fed first from a hidden patch in their flock, were characterized by lower out-strength compared to the other individuals in their flock (first-feeder birds: 4.62 ± 4.74, other flock-mates: 8.63 ± 7.82, *Z* = 2.34, *P* = 0.017), so they followed others less frequently in the flock than other birds (Fig. 2A). On the other hand, these individuals did not elicit more followings in the pre-training period than other birds in their flock, i.e. in-strength of the first-feeders did not differ from that of their flock-mates (first-feeder birds: 6.91 ± 5.69, other flock-mates: 8.17 ± 6.80, *Z* = 0.48, *P* = 0.633; Fig. 2B).

## Discussion

In this study we tested how previously established social connections between house sparrows affected the discovery of hidden food patches, and showed that information about the first-discovered food patch transmitted through the established social networks in the flocks. This result is in line with previous works that demonstrated the importance of social connections in the context of group foraging in various animal species^1, 19, 21^, and stresses the importance of social connections to knowledgeable or experienced individuals within a group when accessing and exploiting novel food sources. However, this pattern was true only for the first-discovered patches, while at the second-discovered patches social attraction was not related to the measured social connections between individuals. This difference arose despite the very small distance between the two patches (0.5–1.5 m) which were similarly accessible and profitable to the birds, reflecting that social reinforcement processes operated in individual decision-making during social foraging even at this spatial scale^2^. Presumably as a consequence of differences in both the time of first discoveries and the level of social attraction at the two food patches, first-discovered patches were exploited by more individuals and also to a greater extent by the end of the trial, which possibly was the cause for a higher number of aggressive interactions at these patches. This may indicate that social attraction caused birds to perceive the first patch as having higher quality than the second one, because of either a lower perceived predation threat when they foraged in close-knit groups or facilitation in foraging due to the quicker dismantling of the millet spray.

Another interesting result is that first-feeder birds were characterized by lower out-strength, i.e. these individuals followed others at a lower frequency compared to their flock-mates. This implies that first-feeder birds were socially more indifferent compared to others, thus less affected by social attraction effects. One could speculate that first feeder individuals could also be more explorative than their flock-mates and/or characterized by reduced neophobia, which idea is in accordance with recent works where personality differences between individuals were found to substantially affect the use of social information and group decision-making^13, 48, 49^. Similarly to these studies, where proactive individuals were found to rely less on social information and have generally weaker social bonds, we found that first-feeder birds were less motivated to follow others in their flock. Thus, our finding supports the idea that social indifference and exploratory behaviour are often positively related individual traits^49^.

House sparrows are a highly adaptable and opportunistic species for which discovering and exploiting novel food sources is crucial to survival. In areas where their range is still expanding, they have been shown to be bolder and to exhibit lower levels of neophobia in an asocial context^50, 51^. However, house sparrows usually forage in flocks, which are often composed of individuals with phenotypic differences in boldness, social indifference and also presumably in following behaviour. The difference in phenotypic composition of a group has been recently emphasized as having a potential impact on fitness^52, 53^, also in this species during social foraging^54^. In our study, the measured social connections within the flocks were likely to be robust (similarly as in ref. 38), and also variable enough to affect the order of discovery of the hidden food source, along with individual characteristics such as sex and foraging activity, a few days after the social network was first assessed. Our results therefore also implicate that the discovery of new food sources, a fundamental aspect for the survival of house sparrows, is strongly influenced by the presence of phenotypic polymorphisms within the flocks^48^. An important question that consequently arises is to what extent the social environment within a house sparrow flock could influence the fitness of its members, or even favour certain group compositions or polymorphisms combinations compared to others^55, 56^. This would be particularly interesting to investigate in a landscape of novel and ephemeral food sources and unexpected threats, such as the environment where house sparrows and other invasive species usually thrive^50^.

In the studied sparrow flocks, patch discovery could be a stochastic event^12^, but as soon as one patch was discovered by a socially less sensitive bird and then utilized by more and more individuals, it was likely to remain the major food source for many individuals throughout the trial. Our results provide experimental evidence for the influential effect of social connections on foraging decisions in the presence of multiple food patches in the house sparrow. We propose that similar studies should investigate how acquisition rate at one food source may directly affect and lead to the change of the acquisition rate at other food sources (e.g. as patch discovery become faster at one patch, discovery rate decelerates at another patch). Also, using a Bayesian estimation of decision making rule proposed by Arganda and collaborators^3^ additional experiments could further scrutinize the consequences of subsequent foraging choices of individuals on resource exploitation at different food sources, linking animal social foraging and individual decision making to the framework of information cascades^57, 58^.

## Electronic supplementary material

SupplementaryInformation
