# Supplementary material for: The effect of social connections on the discovery of multiple hidden food patches in a bird species
Source: Sci Rep. 2017 Apr 11;7:816. doi: 10.1038/s41598-017-00929-8 (PMC5429748; doi:10.1038/s41598-017-00929-8)
Supplement: Supplementary file 1 — SupplementaryInformation [file 41598_2017_929_MOESM1_ESM.pdf]

Supplementary Information

The effect of social connections on the discovery of multiple hidden food patches in a bird species

Zoltán Tóth, Beniamino Tuliozi, Davide Baldan, Herbert Hoi and Matteo Griggio

Tutor flocks

Two weeks prior to the experiment, two groups of four individuals (two males and two females) were allocated into two outdoor aviaries (3.9 × 1.9 × 3 m; 'tutor aviaries' henceforward). These aviaries were equipped with a roosting tree, several perches, nest boxes and a water basin. On the floor two same sized brown cardboard boxes (33 × 21 × 12 cm) were placed, equidistant to the roosting tree and perches, about 0.5 m from each other. The boxes were open only on one of the long sides opposite to the roosting site. The only food source in these aviaries was approx. 60 g of millet spray provided daily under one of the boxes, anchored on an inner side so that the food was only visible and accessible when the birds approached the box from the front. On the top of the box containing food a small coloured marking (a 5 cm diameter circle; for a similar approach, please see [1]) was placed and alternated between boxes on consecutive days. The colour of the markings, light blue and magenta, differed between the two tutor aviaries. Before food was added every morning, the boxes were temporarily removed with any leftovers from the aviary, and the floor around the boxes was carefully cleaned. Then, both the empty box and the one hiding the food was put back into the aviaries, and the coloured marking was always associated with the box containing the food so tutor individuals could rely only on the coloured marking and on approaching the boxes to identify the presence of food. These aviaries were used to train informed individuals for the experimental flocks.

Experimental flocks

The experimental flocks consisted of ten adult individuals (five males and five females) randomly chosen from the eight unisex outdoor aviaries. Each individual was used once during the study. The experimental individuals were transferred into an experimental aviary and individually banded with metallic and coloured rings. To facilitate the identification of birds from video recordings, the crown feathers of all the individuals were painted with non-toxic coloured markers (Deco painter matt, Marabu GmbH & Co. KG, Germany). Tarsus, wing and tail length (to the nearest 0.1 mm) were measured as well as body mass before and after the experiment (to the nearest 0.1 g). The experimental aviary (2.8 × 2.7 × 2.1 m) was equipped with a roosting tree, several perches, and a water basin situated at the back of the aviary. A single feeder was situated at the center of the aviary on a small platform approx. 10 cm off the ground, and served as the main food source. Commercial food for granivorous passerines was provided on the feeder, but the amount differed according to the experimental design. In the front side of the experimental aviary, similarly to the tutor aviaries, four same sized brown cardboard boxes (identical to the ones placed into the tutor aviaries) were placed on the floor, about 20 cm distant one another. These boxes were only open on the side opposite to the roosting site and were fixed on the floor.

Three webcams (Microsoft Lifecam Studios, model Q2F00015) were placed inside the aviaries throughout the entire experimental period, one recording the activity at the central feeder, and the remaining two positioned in front of those two cardboard boxes (approx. 30 cm distant) which were *a priori* randomly selected for hiding the food during the trial. Other than natural light from different windows, artificial light was also provided with 12:12 h light:dark periods (07:00-19:00). The experimental indoor aviary was maintained at a temperature of about 20 Celsius degrees. At the formation of the experimental group, birds were allowed to become familiar with the environment of the experimental aviary for 1 day, during which food was provided *ad libitum*

on the central feeder. The evening before the onset of the trial the feeder was removed and the floor carefully cleaned from seeds.

Network-based diffusion analysis (NBDA)

NBDA was initially developed by Franz & Nunn [2] and extended by Hoppitt et al. [3] (for additional extensions see also 4, 5-7]. We used the order of acquisition diffusion analysis (OADA) variant of NBDA [3], where the model is fit on the order of individual acquisitions, thus measures the relative rate at which individuals acquire the trait. OADA has the advantage that it is insensitive to the shape of the baseline function, and is recommended to be used if the baseline rate of acquisition changes over time [3]. However, a weakness of OADA is that this method can detect social transmission only if it results in substantial differences between the rates of acquisition by which individuals acquire the trait [3]. In a standard OADA, the baseline rate of acquisition is unspecified with the assumption that each diffusion has its own baseline rate. Alternatively, different diffusions or tasks may be included in the same stratum, in which case they are treated as a single diffusion with zero connections among individuals from different diffusions and the same baseline rate function can be assumed in all diffusions within each stratum. Stratifying by food patch in our study also allowed us to estimate different social transmission parameters for each stratum, i.e. for each food patch in the flocks. With this set-up, the potential influence of social connections in homogeneous networks on patch discovery could also be tested. Individual-level variables influencing the rate at which an individual acquires a trait can be incorporated into an OADA using an additive model:

$$R_{i,l}(n) = \left(1 - z_i(n)\right)\left(s_l \sum_{j=1}^{N} (\alpha_{i,j}\, z_j\,(n)) + (1 - s_l)\exp\left(\sum_{k=1}^{V} \beta_{k,} x_{k,i}\right)\right), \quad (1)$$

or individual-level variables can be incorporated using a multiplicative model:

$$R_{i,l}(n) = \left(1 - z_i(n)\right)\left(s_l \sum_{j=1}^{N} (\alpha_{i,j}\, z_j(n)) + (1 - s_l)\right)\exp\left(\sum_{k=1}^{V} \beta_k x_{k,i}\right), \qquad (2)$$

where $R_{i,l}(n)$ is individual *i*'s relative rate of acquisition of the trait immediately prior to the *n*th acquisition event in stratum *l*, $z_i(n)$ is the status of individual *i* prior to the *n*th acquisition event, $s_l \geq 0$ is a parameter determining the rate of social transmission between individuals per unit of network connection in stratum *l* ($s_l = 0$ indicates that all acquisition is by asocial means in stratum *l*), $\alpha_{i,j}$ is the network connection leading from individual *j* to *i*, $z_j(n)$ is the status of *j* prior to the *n*th acquisition event (1 indicates informed and 0 indicates naïve), *N* is the number of individuals, β_k is the coefficient determining the effect of variable *k*, $x_{k,i}$ is the value of variable *k* for individual *i*, and *V* is the number of individual level variables in the model [3,8].

Table S1. Observed foraging events in the house sparrow flocks during the pre-training period. Identified foraging events represent those observed events at the central feeder for which all participants were successfully identified. The total number of visits for an individual was calculated as the sum of the number of arriving at the central feeder alone, by following a flock-mate and in groups without a specific initiator; the flock-level measure of this variable was obtained by summing the individual-level data across all birds in a given flock.

Flock	# of identified foraging events	Identification accuracy (%)	# of followings	Total # of visits
1	1419	97.26	678	1855
2	2093	92.32	1242	2704
3	426	93.83	101	512
4	2362	87.35	543	3471
5	1420	82.80	758	1892
6	1645	82.41	937	2229
7	1578	97.23	730	2068
8	1143	98.79	394	1398
9	880	97.56	351	1108

10	1014	99.12	217	1175

Table S2. Type and relative support of the fitted 72 models. Present: social model (i.e. social transmission is present at least at one patch), Absent: asocial model (i.e. no social transmission); Same *s* at the patches: social transmission rate is the same at the two patches, Different *s* at the patches: social transmission rate is different at the two patches, *s* only at patch 1: social transmission rate is estimated only at the first-discovered patch, *s* only at patch 2: social transmission rate is estimated only at the second-discovered patch; F: fitted with following-based networks, H: fitted with homogeneous networks; ILV: individual-level variable (i.e. 'sex', 'age', or 'feeding activity'). Models in the 'best models' set (i.e. models fitted with the following-based networks and within 4 ΔAICc to the best-fitting model) are written in bold.

Model order	Social transmission	Model category	Type	ILV	AICc	ΔAICc	w_{Akaike} (%)
**1**	**present**	***s* only at patch 1**	**F**	**sex, feeding activity**	**1037.11**	**0.00**	**0.23**
**2**	**present**	***s* only at patch 1**	**F**	**sex, age, feeding activity**	**1037.81**	**0.70**	**0.16**
**3**	**present**	**different *s* at the patches**	**F**	**sex, feeding activity**	**1038.05**	**0.94**	**0.14**
**4**	**present**	**different *s* at the patches**	**F**	**sex, age, feeding activity**	**1039.00**	**1.89**	**0.09**
5	present	same *s* at the patches	H	sex	1040.10	2.99	0.05
6	present	same *s* at the patches	H	-	1040.41	3.30	0.04
**7**	**present**	***s* only at patch 1**	**F**	**feeding activity**	**1040.80**	**3.69**	**0.04**
**8**	**present**	***s* only at patch 1**	**F**	**age, feeding activity**	**1041.08**	**3.97**	**0.03**
9	present	different *s* at the patches	H	sex	1041.66	4.55	0.02
10	present	same *s* at the patches	H	sex, feeding activity	1041.74	4.63	0.02
11	present	same *s* at the patches	H	feeding activity	1041.85	4.74	0.02
12	present	different *s* at the patches	H	-	1041.94	4.83	0.02

13	present	same *s* at the patches	H	sex, age	1042.25	5.13	0.02
14	present	different *s* at the patches	F	feeding activity	1042.43	5.32	0.02
15	present	same *s* at the patches	H	age	1042.50	5.39	0.02
16	present	different *s* at the patches	F	age, feeding activity	1042.89	5.78	0.01
17	present	different *s* at the patches	H	sex, feeding activity	1043.37	6.25	0.01
18	present	different *s* at the patches	H	feeding activity	1043.45	6.34	0.01
19	present	different *s* at the patches	H	sex, age	1043.82	6.71	0.01
20	present	same *s* at the patches	H	sex, age, feeding activity	1043.92	6.81	0.01
21	present	same *s* at the patches	H	age, feeding activity	1044.00	6.89	0.01
22	present	different *s* at the patches	H	age	1044.02	6.91	0.01
23	present	same *s* at the patches	F	sex, feeding activity	1045.24	8.12	0.00
24	present	different *s* at the patches	H	sex, age, feeding activity	1045.58	8.47	0.00
25	present	same *s* at the patches	F	sex, age, feeding activity	1046.81	9.70	0.00
26	present	different *s* at the patches	H	age, feeding activity	1048.06	10.94	0.00
27	present	same *s* at the patches	F	feeding activity	1048.40	11.28	0.00
28	present	same *s* at the patches	F	age, feeding activity	1049.79	12.67	0.00
29	present	*s* only at patch 1	F	sex, age	1050.37	13.26	0.00
30	present	*s* only at patch 1	F	sex	1050.81	13.69	0.00
31	present	*s* only at patch 1	H	sex	1050.82	13.71	0.00
32	present	*s* only at patch 1	H	-	1050.96	13.85	0.00
33	present	*s* only at patch 1	H	feeding activity	1051.68	14.57	0.00

34	present	*s* only at patch 1	H	sex, feeding activity	1051.80	14.68	0.00
35	present	*s* only at patch 1	H	age	1052.29	15.18	0.00
36	present	*s* only at patch 1	H	sex, age	1052.31	15.20	0.00
37	present	*s* only at patch 1	F	age	1052.56	15.44	0.00
38	present	different *s* at the patches	F	sex, age	1052.56	15.45	0.00
39	present	different *s* at the patches	F	sex	1052.96	15.84	0.00
40	present	*s* only at patch 1	H	age, feeding activity	1053.38	16.27	0.00
41	present	*s* only at patch 1	F	-	1053.57	16.46	0.00
42	present	*s* only at patch 1	H	sex, age, feeding activity	1053.60	16.49	0.00
43	present	different *s* at the patches	F	age	1054.71	17.59	0.00
44	present	different *s* at the patches	F	-	1055.69	18.58	0.00
45	present	*s* only at patch 2	H	-	1056.10	18.99	0.00
46	present	*s* only at patch 2	H	sex	1056.55	19.43	0.00
47	present	*s* only at patch 2	H	feeding activity	1056.56	19.45	0.00
48	present	*s* only at patch 2	H	sex, feeding activity	1057.31	20.20	0.00
49	present	*s* only at patch 2	H	age	1057.97	20.85	0.00
50	present	same *s* at the patches	F	sex, age	1058.08	20.97	0.00
51	present	same *s* at the patches	F	sex	1058.09	20.98	0.00
52	present	*s* only at patch 2	H	sex, age	1058.50	21.38	0.00
53	present	*s* only at patch 2	H	age, feeding activity	1058.66	21.55	0.00
54	present	same *s* at the patches	F	age	1059.32	22.21	0.00
55	present	*s* only at patch 2	H	sex, age, feeding activity	1059.46	22.34	0.00
56	present	same *s* at the patches	F	-	1059.52	22.40	0.00

57	absent	-	-	feeding activity	1063.62	26.51	0.00
58	absent	-	-	-	1064.37	27.26	0.00
59	absent	-	-	sex, feeding activity	1064.55	27.44	0.00
60	absent	-	-	sex	1064.95	27.84	0.00
61	absent	-	-	age, feeding activity	1065.05	27.94	0.00
62	absent	-	-	age	1065.11	28.00	0.00
63	absent	-	-	sex, age	1065.81	28.70	0.00
64	present	*s* only at patch 2	F	feeding activity	1065.83	28.72	0.00
65	absent	-	-	sex, age, feeding activity	1066.02	28.91	0.00
66	present	*s* only at patch 2	F	-	1066.53	29.42	0.00
67	present	*s* only at patch 2	F	sex, feeding activity	1066.82	29.71	0.00
68	present	*s* only at patch 2	F	sex	1067.17	30.05	0.00
69	present	*s* only at patch 2	F	age, feeding activity	1067.33	30.22	0.00
70	present	*s* only at patch 2	F	age	1067.33	30.22	0.00
71	present	*s* only at patch 2	F	sex, age	1068.09	30.98	0.00
72	present	*s* only at patch 2	F	sex, age, feeding activity	1068.36	31.25	0.00

Table S3. Relative supports for the OADA models fitted separately at the first- and second-exploited food patches. The number of models in each category is written in brackets; values in bold indicate the best supported category at each patch. Relative support was calculated by summing Akaike weights across the set of models. The 'No ILV' models are those which did not include any individual-level variables (i.e. 'sex', 'age', or 'feeding activity').

Food patch	Asocial models	Models with social transmission		
First-discovered patch	0.03% (8)	99.97% (15)	**Multiplicative**	**80.77% (7)**
			Additive	17.39% (7)
			No ILV	1.81% (1)
Second-discovered patch	34.93% (8)	65.07% (15)	**Multiplicative**	**38.00% (7)**
			Additive	27.06% (7)
			No ILV	<0.01% (1)

References

1. Morand-Ferron, J., Hamblin, S., Cole, E. F., Aplin, L. M., & Quinn, J. L. Taking the operant paradigm into the field: associative learning in wild great tits. *PloS ONE*, **10**(8), e0133821 (2015).
2. Franz, M., & Nunn, C. L. Network-based diffusion analysis: a new method for detecting social learning. *Proc. R. Soc. Lond. B* **276**(1663), 1829–1836 (2009).
3. Hoppitt, W., Boogert, N. J., & Laland, K. N. Detecting social transmission in networks. *J. Theor. Biol.* **263**(4), 544–555 (2010a).
4. Atton, N., Hoppitt, W., Webster, M. M., Galef, B. G. & Laland, K. N. Information flow through threespine stickleback networks without social transmission. *Proc. R. Soc. Lond. B* **279**(1745), 4272–4278 (2012).
5. Farine, D. R., Spencer, K. A., & Boogert, N. J. Early-life stress triggers juvenile zebra finches to switch social learning strategies. *Curr. Biol.* **25**(16), 2184–2188 (2015b).
6. Atton, N., Galef, B. J., Hoppitt, W., Webster, M. M., & Laland, K. N. Familiarity affects social network structure and discovery of prey patch locations in foraging stickleback shoals. *Proc. R. Soc. Lond. B*. **281**(1789), 20140579 (2014).
7. Webster, M. M., Atton, N., Hoppitt, W. J., & Laland, K. N. Environmental complexity influences association network structure and network-based diffusion of foraging information in fish shoals. *Am. Nat*. **181**(2), 235–244 (2013).
8. Hoppitt, W., & Laland, K. N. *Social learning: an introduction to mechanisms, methods, and models* (eds Princeton University Press) (Princeton 2013).
